# Simulated or “Phantom” Tumors

**Published:** 1854-11

**Authors:** 


### RECORD OF MEDICAL SCIENCE.

*Simulated or "Phantom" Tumors.* (Cases under the care of Dr. Addison and Dr. Gull.)—Among the circumstances which combine to make the investigations and diagnosis of abdominal tumors difficult, is the existence of a class in which the symptoms are so changeable that it becomes almost impossible to decide whether or not any tumor does exist. The signs are present one day, entirely absent on another, then present again, in a most perplexing manner. Every practitioner of experience must have met with such puzzling cases; but to those who have not, it would be impossible to convey any idea of the degree to which they sometimes simulate real tumors. Dr. Bright, in his papers on Abdominal Tumors, in the Guy's Hospital Reports, mentions a case in which, in an hysterical woman, the Surgeon had been induced to attempt ovariotomy, believing that an ovarion cyst was present. The incision having been made, no tumor whatever could be found, and the operator was obliged to desist. The woman fortunately recovered, and the tumor at a subsequent period again made its appearance.

One of the earliest allusions to this deceptive class of cases was, we believe, by Dr. Bright; and in the wards of Guy's Hospital they have since been the subject of much investigation. Our own knowledge of them has been chiefly derived from the clinical observations of Drs. Addison and Gull, under whose care several very instructive cases have occurred during the last few years. To the latter gentleman it is, we believe, that the affection is indebted for its very appropriate name of "phantom tumor." We shall attempt in the following sentences a short summary of such facts as have been made out respecting them, but shall not occupy space with the details of cases, as the disease is one in which the prominent symptoms, from being essentially unreal, are interesting rather to the manipulator at the bed-side than to the reader of notes. Dr. Bright's allusion to the subject, to which we have referred, is as follows. In speaking of reported cases of disappearance of ovarian cysts, that experienced Physician states:—"It is even possible that a certain number of these cases may be set down as instances of erroneous diagnosis; for there is no question that the diagnosis is not always obvious. There is one class of cases more particularly liable to lead the unwary and inexperienced into error respecting the disappearance of an abdominal tumor;—I mean cases of hysterical distention of the bowels; for, although the swelling in these cases is essentially tympanitic, yet occasionally, from the singular way in which the intestines are partially distended, and remain so for days and weeks at a time, they sometimes give completely the forms of tumors; and sometimes even indistinct fluctuation may arise from fluid fæces, or even from the coexistence of a distended bladder; and sometimes the large accumulation of hardened fæces has led to a belief of a more solid tumor." To state them *seriatim*, we have then the following, as the chief conditions on which these variable tumors may depend. 1. Distention of the bladder. 2. Solid fæcal accumulations. 3. Irregular contractions of the intestine at two points, and distention of the intervening portion, with flatus or with fluid fæces. 4. Spasmodic rigidity of a part of the abdominal parietes. It may, perhaps, seem almost superfluous to add the last, but practically, it is one of the most frequent sources of deception. An hysterical patient is quite capable of making a circumscribed portion of the abdominal wall rigid and hard, while the rest remains comparatively flaccid; and even in a person of calm nervous system the same condition may be produced by an instinctive reflex act, for the protection of a part of the belly which is tender on pressure. The recti muscles are peculiarly apt to the seat of these contractions, which may, however, also occur in the lateral regions of the abdomen. It is rare, perhaps, for any one of the above mentioned causes to exist singly and uncomplicated by any of the others. Neither of the first two, indeed, unless exaggerated by one or other of the latter could properly rank as a "phantom" tumor. Hardened masses of fæces are probably, however, the most frequent of the exciting causes of the affection. By the irritation produced by their lodgment, the intestines are made to contract irregularly, and local tenderness is also induced, which latter, in its turn, acts as an excitant, in producing reflex rigidity of a part of the abdominal parietes. It has been observed of phantom tumors, that they are much more frequent on the right than the left side, and that not rarely there are present in connexion with them indications of renal irritation. Both of these circumstances are probably to be explained by reference to the facilities afforded by the cœcum and ascending colon for the delay and accumulation of scybalous fæces. The period of early adult life would appear to be the one most liable to the development of this chain of symptoms. The simulated tumor in question is by no means met with only in the female sex, some of the most marked examples of it that we have seen having been in young men. As it regards treatment, that should of course be modified according to the peculiar circumstances of the case. A brisk purgative will probably be a remedy almost always useful, and afterwards a course of nervine tonics, or perhaps of anti-spasmodics, may be exhibited with benefit. The chief importance of the cases is in the lesson they convey as to the necessity for great caution before pronouncing positively as to the existence of an abdominal tumor. The Surgeon should always be content, in doubtful cases, to examine his patient, on several separate occasions, before venturing an opinion. In most cases, probably, the careful employment of percussion and palpation will be competent to decide the question correctly; but if there be the least doubt remaining, the diagnosis should be deferred until, after the free action of a purgative, a second examination has been instituted.

We have introduced the above remarks in this part of the series, among the examples of tumors resulting from accumulation of inflammatory products, because it is for such that these fictitious enlargements will generally be mistaken. Cases of typhlitis are perhaps those with which, more especially, they are likely to be confounded, and, next to them, tumors springing from the kidney or abscesses in that organ.—*Medical Times & Gazette.*

